# Inhibition of TORC1 signaling and increased lifespan: gained in translation?

**DOI:** 10.18632/aging.100560

**Published:** 2013-05-21

**Authors:** Charalampos Rallis, Jürg Bähler

**Affiliations:** Department of Genetics, Evolution & Environment and Institute of Healthy Ageing, University College London, London WC1E 6BT, United Kingdom

Dietary restriction (DR) is a well-known intervention to slow down the aging of cells and organisms. Although the molecular mechanisms by which DR prolongs lifespan are poorly understood, the Target of Rapamycin Complex 1 (TORC1) signaling pathway that controls protein translation, autophagy and mitochondrial function, among other growth-related processes, is considered a main player mediating DR effects in diverse model organisms. Inhibition of TORC1 by nutrient limitation or by drugs prolongs lifespan [1], and reduced protein translation has been linked to increased lifespan in several organisms [2].

Fission yeast is emerging as a complementary model system to budding yeast for cellular ageing. Caffeine and rapamycin treatment trigger a range of phenotypes relating to TORC1 inhibition in fission yeast: gene expression reprogramming, inhibition of global protein translation, reduced cell growth, and extension of chronological lifespan [3]. Rapamycin and caffeine differentially affect these conserved, TORC1-dependent processes. For example, caffeine inhibits global protein translation much more than does rapamycin. Combination of the two drugs often augments the phenotypes observed. Drug synergies utilised to either effectively block a sole pathway, or a combination of pathways, are promising for aging studies and the treatment of age-related diseases. For example, combination of low doses of rapamycin and myriocin enhances autophagy, mitochondrial function, AMP kinase pathway activity and genomic stability in budding yeast [4].

How are TORC1 activity and global protein translation associated with the aging process? Recent results from mammalian cell lines indicate that constitutively active TORC1 signaling augments overall protein synthesis by increasing the speed of ribosomal elongation along transcripts [5]. Notably, this increased translational elongation leads to decreased translational fidelity, possibly by promoting misincorporation of amino acids and compromising the co-translational folding of polypeptides. Rapamycin treatment restores the high quality of newly synthesized proteins by slowing the rate of ribosomal elongation, allowing enhanced quality control [5]. In fission yeast, rapamycin prolongs the chronological lifespan of non-dividing, quiescent cells only when administered to cells that are still proliferating, while rapamycin treatment of cells that have entered quiescence does not affect their lifespan [3]. This intriguing phenomenon highlights an information flow from cellular growth, when TORC1 is active, to cellular quiescence, when it is not. It is plausible that this phenomenon is caused rapamycin increasing protein quality at the expense of protein quantity [5]. A lowered translational activity during cell growth could reduce waste and rogue proteins that would otherwise compromise the longevity of quiescent cells. Similarly, fission yeast cells that are grown slowly in low glucose medium show increased lifespan during quiescence [6], which also could be explained by the increased translation fidelity through decreased TORC1 activity. Both in fission yeast and mammalian cell lines, rapamycin mediates its inhibitory effect on protein translation via ribosomal S6 kinases (S6K) [3,5], and S6K is key in restoring translational fidelity upon rapamycin treatment [5].

Does dietary restriction have the same effect on protein quality at the organismal level, and how much does this effect contribute to longevity? Moreover, as TORC1 signaling also controls gene transcription, does over-activation of this pathway cause similar fidelity issues for transcripts as for proteins? The hyperfunction theory of aging postulates that the proximal cause of aging is not the accumulation of random molecular damage but overactive cellular functions [7]. While increased random damage of existing molecules might not significantly contribute to aging, is it possible that TORC1-driven hyperfunction leads directly to the increased synthesis of rogue proteins that cause pathologies and compromise longevity? If cellular hyperfunction underlies the aging process, what would be the contribution of any associated defects in protein homeostasis in the whole process? A grand challenge in aging research is to define all the factors that affect longevity and uncover any universal causes of cellular and organismal aging. The nutrient-sensing TORC1 signaling pathway seems to be a key contributor: its inhibition by drugs or dietary restriction reduces protein translation, apparently allowing for better quality control. Accumulation of faulty and mis-folded proteins might thus be reduced, which otherwise would directly contribute to age-related cellular pathologies.
